# Casamiento infantil y salud perinatal en Ecuador, 2015-2020

**DOI:** 10.18294/sc.2023.4325

**Published:** 2023-04-13

**Authors:** Dorian Ospina Galeano, Fadya Asia Orozco, Marcelo Luis Urquia

**Affiliations:** 1 Gerente de Sistemas de Información en Salud. Estudiante, Community Health Sciences (MSc), Rady Faculty of Health Sciences, University of Manitoba, Winnipeg, Manitoba, Canadá. ospinagd@myumanitoba.ca University of Manitoba Community Health Sciences (MSc) Rady Faculty of Health Sciences University of Manitoba Winnipeg Manitoba Canada ospinagd@myumanitoba.ca; 2 Doctora en Salud Pública. Docente Titular, Escuela de Salud Pública, Universidad San Francisco de Quito, Quito, Ecuador. forozco@usfq.edu.ec Universidad San Francisco de Quito Escuela de Salud Pública, Universidad San Francisco de Quito Quito Ecuador forozco@usfq.edu.ec; 3 PhD in Epidemiology. Associate Professor, Department of Community Health Sciences, Rady Faculty of Health Sciences, University of Manitoba, Winnipeg, Manitoba, Canadá. marcelo.urquia@umanitoba.ca University of Manitoba Department of Community Health Sciences Rady Faculty of Health Sciences University of Manitoba Winnipeg Manitoba Canada marcelo.urquia@umanitoba.ca

**Keywords:** Edad Materna, Nacidos Vivos, Estado Civil, Peso al Nacer, Nacimiento Prematuro, Ecuador.

## Abstract

Este estudio buscó estimar la prevalencia y distribución de nacidos vivos de madres menores de 18 años en Ecuador y la asociación entre indicadores perinatales y estado marital materno. A partir de los registros de nacidos vivos obtenidos del Instituto Nacional de Estadísticas y Censos de Ecuador para el período 2015-2020, se estimó la asociación conjunta entre grupos de edad (10-15, 16-17, 18-19 y 20-24 años) y la situación conyugal materna (casada, unión estable y soltera), con bajo peso al nacer, parto pretérmino e inadecuada atención prenatal. La prevalencia de partos de madres menores de 18 años fue del 9,3% y declinó en el periodo de estudio, drásticamente entre las mujeres casadas. La asociación entre estado marital y las variables explicativas dependió de la edad materna. Los resultados más favorables de salud observados entre las madres casadas de 20-24 años, en comparación con las madres solteras, se debilitan o desaparecen entre las menores de edad. Las madres en uniones de hecho experimentaron resultados intermedios entre las mujeres casadas y las solteras.

## INTRODUCCIÓN

La reducción de los embarazos adolescentes y del matrimonio infantil son considerados como objetivos prioritarios para la prevención de gestaciones tempranas y resultados de salud reproductiva adversos en adolescentes^1^. 

El casamiento infantil, definido como una unión formalmente legalizada o de hecho antes de los 18 años de edad, está siendo crecientemente reconocido a nivel global por varias organizaciones y gobiernos como una amenaza a los derechos humanos, especialmente, el de las niñas^2^^,^^3^^,^^4^. Las mujeres se casan antes de los 18 años con mayor frecuencia que los varones, y a edades más tempranas, lo cual refleja desigualdades de género que pueden afectar negativamente la salud, educación y desarrollo autónomo de las mujeres a lo largo de la vida^5^. 

Cada año, aproximadamente 12 millones de niñas se casan antes de los 18 años de edad^5^. Aunque el casamiento de niñas es más prevalente en países de África subsahariana y del sur de Asia^6^^,^^7^, se trata de un fenómeno global, con altas prevalencias observadas en países de América Latina como Brasil^8^ e incluso en países como Canadá y EEUU^9^^,^^10^^,^^11^^,^^12^. 

Las mujeres casadas generalmente exhiben mejores resultados de salud perinatal que las solteras^13^^,^^14^^,^^15^, mientras que las que están en uniones de hecho exhiben resultados intermedios^14^^,^^15^. Se considera que la ventaja del matrimonio adulto puede derivar de una influencia positiva del matrimonio como institución social (por ejemplo, esta hipótesis considera que el casamiento contribuye a la adopción y mantenimiento de actitudes y conductas más saludables y a la prevención de conductas de riesgo); de que la selección de individuos que optan por el matrimonio es más saludables (por ejemplo, esta hipótesis considera que no es el matrimonio en sí sino que los individuos que finalmente se casan tienen características sociodemográficas privilegiadas y, por lo tanto, son más saludables que los que no se casan); o de una combinación de los dos mecanismos^16^^,^^17^. Independientemente del mecanismo, la cuestión de si los beneficios de salud asociados al matrimonio adulto también se extienden a las mujeres menores de edad no ha sido bien estudiada.

La mayor parte de la literatura acerca del matrimonio infantil y sus consecuencias sociales y de salud proviene de estudios llevados a cabo en países de Asia y África, donde la mayoría de los embarazos tempranos ocurre dentro de matrimonios arreglados^5^. Estos estudios relacionan el matrimonio infantil con baja escolaridad de las mujeres, limitada autonomía, violencia doméstica, embarazos no deseados, mayor fertilidad y resultados de salud reproductiva más pobres que en las mujeres que se casaron en la mayoría de edad^18^^,^^19^^,^^20^^,^^21^^,^^22^. Sin embargo, estas asociaciones no pueden ser fácilmente generalizadas al contexto latinoamericano, donde la mayoría de los embarazos ocurre por fuera del matrimonio formal, los nacimientos fuera del matrimonio son crecientemente aceptados, y la mayoría de los matrimonios y uniones de hecho son supuestamente consensuales^23^.

En Ecuador, la mayoría de edad, definida como la edad mínima a partir de la cual una persona ciudadana es considerada capaz de tomar decisiones responsables y ejercer libremente los derechos y responsabilidades en la sociedad, comienza al cumplir los 18 años de edad. En el año 2015, Ecuador modificó el Código Civil pasando a prohibir el matrimonio a menores de 18 años de ambos sexos. Hasta entonces, la edad mínima para contraer matrimonio era de 14 años para los varones y de 12 años para las mujeres^24^. En la década de 2020, el matrimonio infantil se encuentra prohibido solo en nueve países de América Latina y dos del Caribe^25^.

Aunque muchos estudios cuantitativos perinatales suelen agrupar a gestantes menores de 20 años en un solo grupo para sortear limitaciones de tamaño muestral o para facilitar comparaciones con otros grupos de edad, existe una gran heterogeneidad de riesgo dentro de este grupo, la cual refleja la influencia de diversos factores, tales como la nutrición, la menarca y la edad ginecológica^26^. La minoría de edad y el casamiento infantil son entidades socioculturales que adicionalmente resaltan la importancia de distinguir subgrupos de edad entre gestantes menores de 20 años en estudios de salud reproductiva, con el fin de posibilitar un examen más detallado de gradientes de riesgo dentro de las adolescentes y menores de edad, particularmente en lo que respecta a la intersección entre la edad materna y el estado marital.

Para avanzar nuestro conocimiento sobre las complejas relaciones entre el estado marital, la edad materna e indicadores de salud reproductiva, este estudio se centra en un análisis de todos los nacimientos ocurridos en Ecuador desde 2015, año en que el casamiento infantil fue prohibido. Este estudio tiene como objetivos estimar la prevalencia y distribución de nacidos vivos de madres menores de 18 años en Ecuador y la asociación entre indicadores perinatales y el estado marital de mujeres que tuvieron nacidos vivos en los grupos de edad de 10-15, 16-17, 18-19 y 20-24 años. Este estudio proporciona información útil para ayudar a entender las características relacionadas a los estados maritales entre las menores de edad, adolescentes y adultas jóvenes, y las asociaciones con indicadores de salud reproductiva.

## MATERIALES Y MÉTODOS

Se realizó un estudio de corte transversal de base poblacional. Los registros de nacidos vivos de Ecuador, de los años 2015-2020, fueron obtenidos del Instituto Nacional de Estadísticas y Censos (INEC). A partir del año 2015, Ecuador implementó el certificado de nacimiento electrónico en línea a través del Sistema Nacional de Registros Vitales (REVIT), que progresivamente subió de 88 establecimientos asistenciales en 2015, a 569 en 2017 y a 606 en 2020^27^. El formulario físico de nacido vivo continuó usándose en partos no institucionales y en las instituciones que aún no se han integrado al REVIT, y es descargado por las instituciones, completado y devuelto al INEC para su procesamiento^27^. Tanto el formulario electrónico en línea como el formulario físico son compilados y enviados mensualmente a la Dirección de Registros Administrativos (DIRAD) del INEC, donde se procede a la evaluación de calidad y procesamiento de la información. Las inconsistencias en los datos se aclaran con las fuentes de información (Oficinas del Registro Civil y establecimientos de salud), previamente a ser consolidados en las bases de datos por el INEC^27^.

### Población de estudio

Hubo 1.826.456 registros de nacidos vivos en el periodo de estudio. Entre estos, se excluyeron 116.468 (6,4%) por ocurrir antes del año 2015, 51.609 (3,0%) por ser registrados después del 31 de marzo del año siguiente al del nacimiento, y 3.277 (0,2%) por no indicar el año de nacimiento. También se excluyeron 81 (0,004%) nacidos vivos de madres mayores de 49 años y 25.314 (1,5%) de madres de edad desconocida, dejando 1.629.707 nacidos vivos de madres de 10 a 49 años para la estimación de la proporción de madres <18 años entre todas las madres en edad reproductiva.

Para evaluar la relación entre bajo peso al nacer, partos prematuros y atención prenatal de acuerdo al estado marital de madres menores, adolescentes y jóvenes, se excluyeron 877.926 registros de nacidos vivos de madres ≥ 25 años, 7.558 registros de partos múltiples, 5.544 sin información sobre etnicidad o estado marital materno, 20.823 sin información sobre el peso al nacer o la edad gestacional, 860 en los que el peso al nacer excedió en 4 desviaciones estándar de la media de peso para cada sexo y semana de gestación, 4.094 de madres divorciadas, separadas o viudas o sin información sobre lugar de residencia. Algunos registros cumplieron más de un criterio de exclusión. Por lo tanto, la población de estudio para los análisis multivariados de bajo peso y partos pretérmino incluyó 712.902 nacidos vivos de madres de 10 a 24 años, y 710.723 para los análisis de inadecuada atención prenatal, luego de excluir 2.179 registros sin información sobre atención prenatal.

### Medidas

La situación marital de la madre se categorizó en casada legalmente, unión estable, soltera, viudas/separadas/divorciadas y no reportada. Para los análisis descriptivos, cuyo objetivo es determinar la prevalencia de casamiento infantil, los nacidos vivos de madres viudas, separadas y divorciadas se colapsaron con los de madres casadas, dado que habían estado casadas previamente. Sin embargo, debido al bajo número de nacidos vivos de madres viudas/separadas/divorciadas y sin reportar, estos no se incluyeron como grupos de comparación en los análisis multivariados.

La edad de la madre se categorizó en los siguientes grupos: 10-15, 16-17, 18-19, 20-24 y 25-49 años. El grupo de 25-49 años no se incluyó en los análisis multivariados debido al foco en madres menores, adolescentes y jóvenes.

El bajo peso al nacer (<2.500 g) fue subdividido en muy bajo peso (<1.500 g) y moderadamente bajo peso (1.500-2.499 g), siendo el peso normal de ≥ 2.500 g el grupo de referencia. El parto pretérmino (<37 semanas de gestación) fue subdivido en muy (24-31 semanas) y moderadamente pretérmino (32-36 semanas), siendo los partos a término (37 semanas y más) el grupo de referencia. 

La atención prenatal se categorizó en adecuada e inadecuada, basada en el *Revised Graduated Prenatal Care Utilization Index* (Revised-GINDEX)^28^, que combina información de cantidad de visitas prenatales y edad gestacional.

### Análisis estadístico

Para los análisis descriptivos tendientes a estimar la prevalencia de madres <18 años, según estado marital y características sociodemográficas, se usaron proporciones expresadas en porcentajes. Se usó como denominador a todos los nacidos vivos de madres de 10-49 años.

Para evaluar las asociaciones entre bajo peso, partos prematuros y atención prenatal con el estado marital de la madre, la población de estudio se limitó a madres de 10 a 24 años. La regresión logística multinomial se usó para modelar los dos niveles de bajo peso (muy bajo y moderadamente bajo) y de pretérmino (muy y moderadamente), mientras que para la atención prenatal inadecuada (sí versus no) se utilizó regresión logística binomial. Para evaluar la interacción entre los grupos de edad y la situación marital materna, se introdujo en estos modelos un término del producto entre ambas variables. La significación estadística de la interacción fue medida a través del “*Likelihood Ratio Test*”, que compara el modelo “Ln(Odds) = estado marital + grupo de edad + estado marital * grupo de edad” con el modelo más simple “Ln(Odds) = estado marital + grupo de edad”, siendo la hipótesis nula que el modelo más complejo no es más informativo que el modelo simple. Para visualizar la influencia conjunta del estado marital y la edad materna sobre las variables dependientes, los resultados de un mismo modelo se presentaron de dos maneras; i) usando a solteras de 20 a 24 años de edad como único grupo de referencia, y ii) usando a solteras como grupo de referencia dentro de cada grupo de edad, equivalente a un análisis estratificado^29^.

Como variables de ajuste en los modelos se incluyeron sexo del recién nacido (excepto en atención prenatal), alfabetismo (sí, no), primiparidad (sí, no), inmigrante (sí, no), grupo étnico (indígena, afroecuatoriana, mestiza, blanca y otra), área de residencia (rural, urbana) y región de residencia de la madre (Costa, Sierra, Oriente, Insular y otra).

A fin de evitar estimaciones inestables e imprecisas, solamente se reportaron asociaciones basadas en al menos diez eventos en los subgrupos definidos por la intersección entre el estado marital y el grupo de edad materna.

La manipulación de los datos y los análisis estadísticos fueron ejecutados con los programas de análisis estadístico SAS y R. Los gráficos fueron creados con Excel y Prisma. La base de datos de nacidos vivos de Ecuador es de dominio público y por lo tanto su uso no requiere aprobación de un Comité de Ética. 

## RESULTADOS

En Ecuador, entre 2015 y 2020, hubo 1.629.707 nacidos vivos de mujeres entre 10 y 49 años de edad (Tabla 1a y Tabla 1b). Entre ellos, 147.936 (9,1%) correspondieron a madres <18 años, y 36.934 (25%) a madres de entre 10 y 15 años. La proporción de mujeres casadas legalmente creció con la edad materna, aunque fue muy baja entre las <18 años. Sin embargo, un tercio de las que tuvieron hijos entre los 10-15 y 16-17 años de edad estaba en uniones estables. 

**Tabla 1a:** Distribución de nacidos vivos de acuerdo con características de la madre, según edad de la madre. Ecuador, 2015-2020.

Características de la madre	Edad de la madre en años											
	10-15		16-17		18-19		20-24		25-49		Total	
	n	%	n	%	n	%	n	%	n	%	n	%
Total	36.934	2,3	111.002	6,8	167.157	10,3	436.688	26,8	877.926	53,9	1.629.707	100,0
Estado marital												
Casada	349	0,9	2.026	1,8	13.711	8,2	85.914	19,7	376.707	42,9	478.707	29,4
Unión estable	12.719	34,4	39.437	35,5	58.059	34,7	138.380	31,7	198.929	22,7	447.524	27,5
Soltera	23.607	63,9	68.795	62,0	94.136	56,3	207,681	47,6	270,040	30,8	664.259	40,8
Separada, divorciada o viuda	72	0,2	257	0,2	428	0,3	2.555	0,6	28.133	3,2	31.445	1,9
No reportado	187	0,5	487	0,4	823	0,5	2.158	0,5	4.117	0,5	7.772	0,5
Primípara												
Sí	35.824	97,0	99.793	89,9	128.363	76,8	209,958	48,1	172.152	19,6	646.090	39,6
No	1.110	3,0	11.209	10,1	38.794	23,2	226.730	51,9	705.774	80,4	983.617	60,4
Alfabetismo												
Sí	36.609	99,1	110.367	99,4	166.363	99,5	434.230	99,4	869.446	99,0	1.617.015	99,2
No	199	0,5	330	0,3	35	0,2	1.273	0,3	5.969	0,7	8.128	0,5
No reportado	126	0,3	305	0,3	437	0,3	1.185	0,3	2.511	0,3	4.564	0,3
Grupo etnico:												
Indígena	2.696	7,3	8.294	7,5	11.882	7,1	26.581	6,1	49.014	5,6	98,467	6,0
Afroecuatoriana	1.230	3,3	3.037	2,7	4.251	2,5	11.041	2,5	20.145	2,3	39.704	2,4
Mestiza	32.509	88,0	98.430	88,7	148.979	89,1	392.821	90,0	792.846	90,3	1.465.585	89,9
Blanca	240	0,6	634	0,6	974	0,6	2.981	0,7	8.524	1,0	13.353	0,8
No reportado	259	0,7	607	0,5	1.071	0,6	3.264	0,7	7.397	0,8	12.598	0,8
Área de residencia												
Rural	9.654	26,1	28.327	25,5	40.583	24,3	95.776	21,9	183.798	20,9	358.138	22,0
Urbana	27.280	73,9	82.675	74,5	126.574	75,7	340.912	78,1	694.128	79,1	1.271.569	78,0
Región de residencia												
Costa	23.408	63,4	65.599	59,1	96.607	57,8	249.903	57,2	453.396	51,6	888.913	54,5
Sierra	9.693	26,2	36.163	32,6	58.568	35,0	158.994	36,4	371.588	42,3	635.006	39,0
Oriente	3.793	10,3	9.088	8,2	11.747	7,0	27.064	6,2	51.086	5,8	102.778	6,3
Insular	24	0,1	93	0,1	168	0,1	572	0,1	1504	0,2	2.361	0,1
Otra	16	<0,1	59	<0,1	67	<0,1	155	<0,1	352	<0,1	649	<0,1
Nacionalidad ecuatoriana												
Sí	36.304	98,3	109.088	98,3	162.955	97,5	422.387	96,7	848.929	96,7	1.579663	96,9
No	615	1,7	1.883	1,7	4.156	2,5	14.178	3,2	28.692	3,3	49.524	3,0
No reportado	15	<0,1	31	<0,1	46	<0,1	123	<0,1	305	<0,1	520	<0,1

Fuente: Elaboración propia a partir de datos del Instituto Nacional de Estadísticas y Censos de Ecuador.

**Tabla 1b:** Distribución de nacidos vivos de acuerdo con características del parto y del recién nacido, según edad de la madre. Ecuador, 2015-2020.

Características del parto y del recién nacido	Edad de la madre en años											
	10-15		16-17		18-19		20-24		25-49		Total	
	n	%	n	%	n	%	n	%	n	%	n	%
Total	36.934	2,3	111.002	6,8	167.157	10,3	436.688	26,8	877.926	53,9	1.629.707	100,0
Periodo de nacimiento												
2015-2016	13.345	36,1	38.438	34,6	56.300	33,7	141.010	32,3	277.008	31,6	526.101	32,3
2017-2018	13.088	35,4	39.665	35,7	57.866	34,6	153.545	35,2	304.245	34,7	568.409	34,9
2019-2020	10.501	28,4	32.899	29,6	52.991	31,7	142.133	32,5	296.673	33,8	535.197	32,8
Sexo												
Varón	18.676	50,6	57.051	51,4	85.571	51,2	223.920	51,3	447.508	51,0	832.726	51,1
Mujer	18.258	49,4	53.951	48.6	81.586	48,8	212.768	48,7	430.418	49,0	796.981	48,9
Peso al nacer												
Muy bajo (<1.500 g)	494	1,3	1.205	1,1	1.649	1,0	4.077	0,9	10.056	1,1	17.481	1,1
Moderadamente bajo (1.500-2.499 g)	4.006	10,8	10.652	9,6	14.419	8,6	33.236	7,6	66.901	7,6	129.214	7,9
Normal (> 2.499 g)	31.571	85,5	96.655	87,1	147.348	88,1	390.563	89,4	784.534	89,4	1.450.671	89,0
No reportado	863	2,3	2.490	2,2	3.741	2,2	8.812	2,0	16.435	1,9	32.341	2,0
Edad gestational												
Muy prétermino (24-31 semanas)	465	1,3	1.092	1,0	1.472	0,9	3.564	0,8	9.113	1,0	15.706	1,0
Moderadamente prétermino (32-36 semanas)	2.705	7,3	6.727	6,1	9.121	5,5	22.719	5,2	56.012	6,4	97.284	6,0
Normal (37-42 semanas)	32.708	88,6	100.095	90,2	151.91	90,9	399.091	91,4	790.618	90,1	1.474.429	90,5
No reportado	1.056	2,9	3.088	2,8	4.647	2,8	11.314	2,6	22.183	2,5	42.288	2,6
Atención prenatal												
No visitó servicios de salud	876	2,4	1.851	1,7	2.685	1,6	5.595	1,3	8.135	0,9	19.142	1,2
Inadecuada	23.146	62,7	67.077	60,4	96.496	57,7	231.280	53,0	372.653	42,4	790.652	48,5
Adecuada	11.712	31,7	38.604	34,8	62.794	37,6	186.957	42,8	471.330	53,7	771.397	47,3
No reportado	1.200	3,2	3.470	3,1	5.182	3,1	12.856	2,9	25.808	2,9	48.516	3,0
Multiplicidad												
Parto único	36.678	99,3	110.186	99,3	165.621	99,1	431.738	98,9	861.661	98,1	1.605.884	98,5
Multiple	256	0,7	816	0,7	1.536	0,9	4,950	1,1	16.265	1,9	23.823	1,5
Tipo de parto												
Normal	24.633	66,7	75.780	68,3	108.444	64,9	251.638	57,6	398.397	45,4	858.892	52,7
Cesarea	12.242	33,1	35.075	31,6	58.450	35,0	184.432	42,2	478.392	54,5	768.591	47,2
No reportado	59	0,2	147	0,1	263	0,2	618	0,1	1.137	0,1	2.224	0,1
Tipo de profesional que atiende el parto												
Partera o médico tradicional	969	2,6	2.796	2,5	4.217	2,5	9.972	2,3	18.168	2,1	36.122	2,2
Profesional en Salud	35.965	97,4	108.206	97,5	162.940	97,5	426.716	97,7	859.758	97,9	1.593.585	97,8
Lugar del parto												
Institución de salud privada	4.408	11,9	14.920	13,4	26.385	15,8	81.903	18,8	227.806	25,9	355.422	21,8
Institución de salud pública	31.442	85,1	92.922	83,7	135.923	81,3	343.360	78,6	628.951	71,6	1.232.598	75,6
Propia casa u otro lugar	1.084	2,9	3.160	2,8	4.849	2,9	11.425	2,6	21.169	2,4	41.687	2,6

Fuente: Elaboración propia a partir de datos del Instituto Nacional de Estadísticas y Censos de Ecuador.

**Figura 1:**
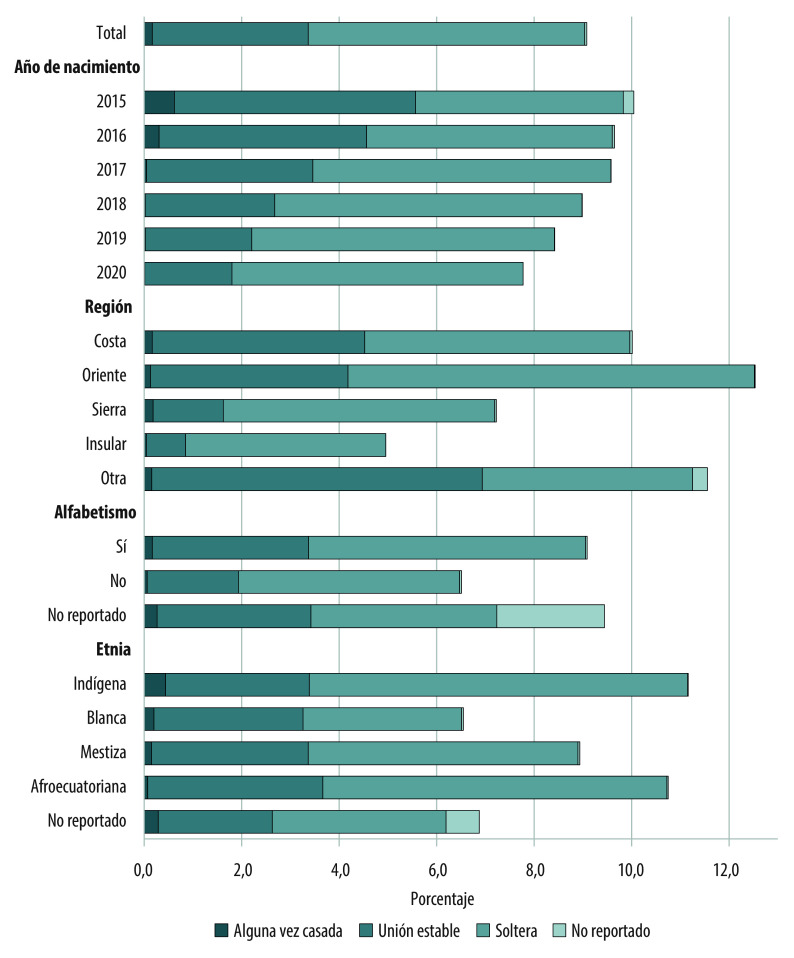
Porcentaje de nacidos vivos de madres < 18 años, según situación conyugal y características sociodemográficas (n=712.902). Ecuador, 2015-2020.

La proporción de madres <18 años disminuyó del 10,0% en 2015 al 7,8% en 2020, principalmente debido a una reducción de madres casadas y en uniones estables (Figura 1). La reducción en madres casadas fue prácticamente completa, del 0,62% en 2015 al 0,01% en 2020. Los nacidos vivos de madres en uniones estables también disminuyeron del 4,95% en 2015 al 1,79% en 2020, una reducción del 64%, mientras que la proporción de nacidos vivos de madres solteras se mantuvo relativamente constante. La prevalencia de nacidos vivos de madres <18 años fue mayor que el promedio del país en las regiones de la Costa y Oriente, así como en madres indígenas y de descendencia afroecuatoriana, con las más altas proporciones de madres solteras.

Fuente: Elaboración propia a partir de datos del Instituto Nacional de Estadísticas y Censos de Ecuador. Nota: Alguna vez casada incluye viuda, separada y divorciada. Otra región incluye áreas no delimitadas y fuera del país.

**Figura 2:**
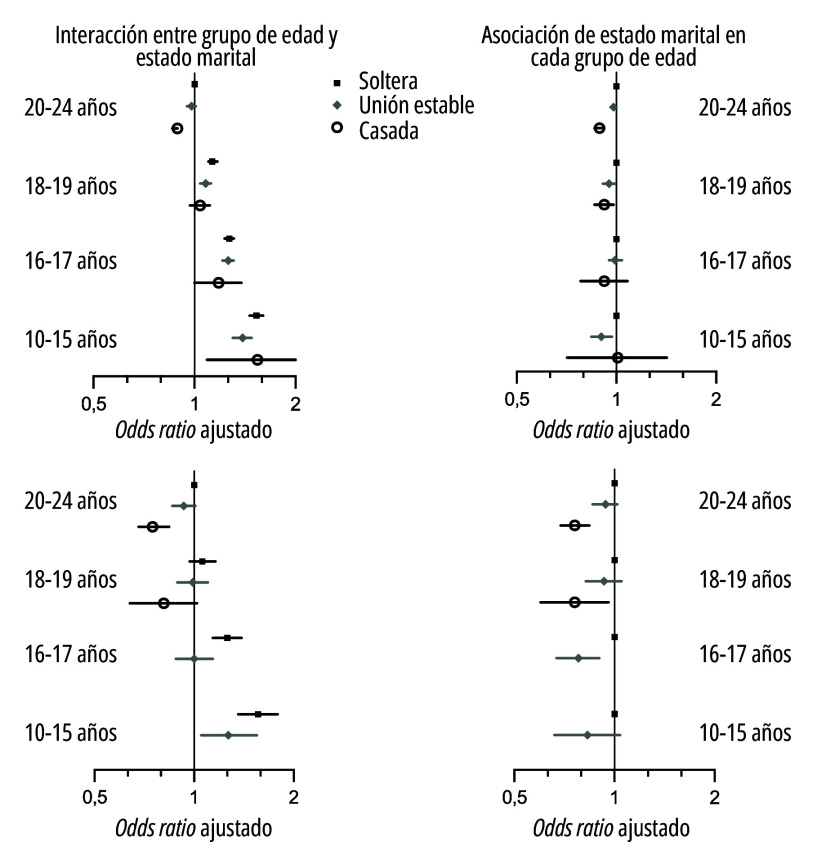
Interacción entre grupo de edad y estado marital y asociación de estado marital en cada grupo de edad, según peso al nacer y grupo de edad de la madre (n=712.902). Ecuador, 2015-2020.

En los análisis multivariados dirigidos a examinar el bajo peso al nacer (Tabla 2 y Figura 2), se observó un claro gradiente de aumento de la proporción de nacidos vivos de moderamente bajo y muy bajo peso a medida que disminuye la edad materna (Figura 2). La asociación entre estado marital y peso al nacer estuvo débilmente modificada por la edad materna (test de interacción: valor de *p*=0,06). Dentro de cada grupo de edad (Figura 2), las madres casadas en los grupos de 18-19 y 20-24 años de edad tuvieron menores chances (*odds ratios*) de moderadamente bajo y muy bajo peso que las madres solteras, pero no se observaron diferencias significativas en los grupos etarios de 10-15 y 16-17 años para moderadamente bajo peso. Debido a la pequeña cantidad de nacidos vivos con muy bajo peso de madres casadas de 10-15 y 16-17 años, no se estimaron estos *odds ratios*. Los nacidos vivos de madres en uniones estables se ubicaron en una posición intermedia entre las mujeres casadas y las mujeres solteras.

**Tabla 2:** Interacción entre grupo de edad y estado marital y asociación de estado marital en cada grupo de edad, según peso al nacer y grupo de edad de la madre (n=712.902). Ecuador, 2015-2020.

Categorías	Grupos de edad (años)	Estado marital	Eventos/nacimientos	%	Interacción entre grupo de edad y estado marital		Asociación de estado marital en cada grupo de edad	
					OR^a^	IC95%	OR^a^	IC95%
Moderado bajo peso al nacer (1.500 – 2.499 g)	20-24	Soltera	14.914/199.548	7,47	1,00*	-	1,00*	-
		Unión estable	9.189/132.807	6,92	0,98	0,95; 1,01	0,98	0,96;1,01
		Casada	5.514/80.814	6,82	0,89	0,86; 0,91	0,89	0,86;0,92
	18-19	Soltera	7.829/90.344	8,67	1,13	1,10;1,17	1,00*	-
		Unión estable	4.349/55.750	7,60	1,06	1,04;1,12	0,95	0,91;0,99
		Casada	1.048/12.699	8,25	1,04	0,97;1,11	0,92	0,80;0,98
	16-17	Soltera	6.378/66.021	9,66	1,27	1,23;1,31	1,00*	-
		Unión estable	3.422/37.642	9,02	1,26	1,23;1,31	0,99	0,91;1,04
		Casada	168/1.838	9,14	1,28	1,00;1,38	0,92	0,78;1,08
	10-15	Soltera	2.542/22.601	11,25	1,53	1,46;1,60	1,00*	-
		Unión estable	1.197/12.217	9,80	1,39	1,30;1,48	0,90	0,84; 0,97
		Casada	37/321	11,53	1,54	1,09;2,17	1,01	0,71; 1,42
Muy bajo peso al nacer (< 1.500 g)	20-24	Soltera	1.540/199.548	0,77	1,00*	-	1,00*	-
		Unión estable	953/132.807	0,72	0,93	0,86;1,01	0,94	0,86;1,02
		Casada	468/80.814	0,56	0,75	0,68;0,84	0,78	0,69;0,84
	18-19	Soltera	726/90.344	0,80	1,06	0,97;1,16	1,00*	-
		Unión estable	416/55.750	0,75	0,99	0,89;1,10	0,93	0,82;1,05
		Casada	77/12.699	0,61	0,81	0,64;1,02	0,76	0,60;0,96
	16-17	Soltera	622/66.021	0,94	1,26	1,14;1,39	1,00*	-
		Unión estable	283/37.942	0,75	1,00	0,88;1,14	0,78	0,67;0,9
		Casada	7/1.838	0,38	-	-	-	-
	10-15	Soltera	257/22.601	1,14	1,56	1,36;1,79	1,00*	-
		Unión estable	114/12.217	0,93	1,27	1,05;1,55	0,83	0,66;1,04
		Casada	0/321	-	-	-	-	-

Fuente: Elaboración propia a partir de datos del Instituto Nacional de Estadísticas y Censos de Ecuador ^a^Modelo multivariado ajustado por sexo del recién nacido, alfabetismo, grupo étnico, paridad (primípara, baja multípara, gran multípara), estado migratorio, región y área de residencia de la madre. Basado en modelos de regresión logística multinomial para las categorías de referencia de edad gestacional a término (37 a 42 semanas) y peso normal (≥ 2.500 g). *Valor de referencia.

Fuente: Elaboración propia a partir de datos del Instituto Nacional de Estadísticas y Censos de Ecuador.

**Figura 3:**
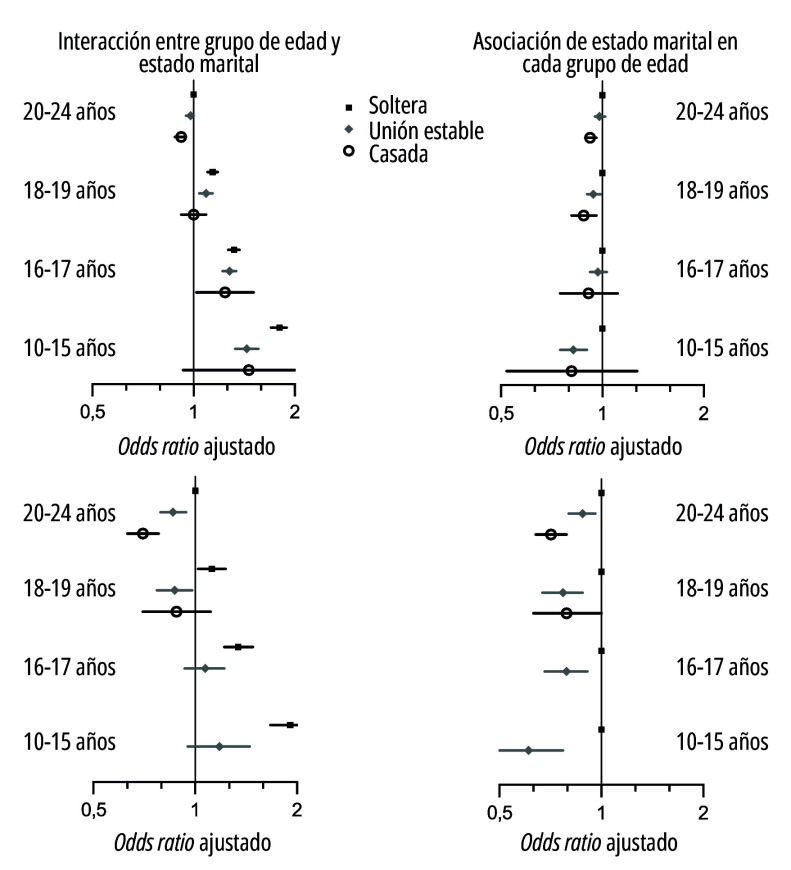
Interacción entre grupo de edad y estado marital y asociación de estado marital en cada grupo de edad, según nacimientos pretérmino y grupo de edad de la madre (n=712.902). Ecuador, 2015-2020.

Un gradiente similar de aumento de partos pretérmino con disminución de la edad materna se observó tanto para las semanas 24-31 como para las semanas 32-36 de gestación (Tabla 3 y Figura 3). La asociación entre estado marital y partos pretérmino se vio modificada por la edad materna (test de interacción: valor de *p*<0,001). Dentro del grupo de edad de 20-24 años, los partos pretérmino fueron menos frecuentes entre las madres casadas, seguidas de las madres en uniones estables y más altos entre las solteras (Tabla 3 y Figura 3). Este patrón fue más marcado para las semanas 24-31 de gestación. Sin embargo, este patrón no se observó entre las madres de 18-19 años y entre las menores de edad. Las madres en uniones estables tuvieron menor frecuencia de partos muy pretérmino que las solteras en todos los grupos de edad. Debido a la virtual inexistencia de partos muy pretérmino entre las madres casadas <18 años, las razones de probabilidad (*odds ratio*) no se pudieron computar. 

**Tabla 3:** Interacción entre grupo de edad y estado marital y asociación de estado marital en cada grupo de edad, según nacimientos pretérmino y grupo de edad de la madre (n=712.902). Ecuador, 2015-2020.

Categorías	Grupos de edad (años)	Estado marital	Eventos/nacimientos	%	Interacción entre grupo de edad y estado marital		Asociación de estado marital en cada grupo de edad	
					OR^a^	IC95%	OR^a^	IC95%
Nacimiento moderadamente pretérmino (32-36 semanas)	20-24	Soltera	9.929/199.548	4,98	1,00*	-	1,00*	-
		Unión estable	6.233/132.807	4,69	0,98	0,95; 1,01	0,98	0,95; 1,02
		Casada	3.814/80.814	4,72	0,92	0,88; 0,95	0,92	0,89; 0,96
	18-19	Soltera	4.887/90.344	5,41	1,14	1,10; 1,18	1,00*	-
		Unión estable	2.730/55.750	4,90	1,09	1,04; 1,14	0,94	0,90; 0,99
		Casada	620/12.699	4,88	1,00	0,92; 1,09	0,88	0,81; 0,96
	16-17	Soltera	4.015/66.021	6,08	1,32	1,27; 1,37	1,00*	-
		Unión estable	2.093/37.942	5,52	1,28	1,22; 1,34	0,97	0,92; 1,03
		Casada	107/1.838	5,82	1,24	1,02; 1,51	0,91	0,75; 1,11
	10-15	Soltera	1.767/22.601	7,82	1,80	1,70; 1,89	1,00*	-
		Unión estable	731/12.217	5,98	1,44	1,33; 1,56	0,82	0,75; 0,90
		Casada	21/321	6,54	1,46	0,93; 2,28	0,81	0,52; 1,27
Nacimiento muy pretérmino (24-31 semanas)	20-24	Soltera	1.479/199.548	0,74	1,00*	-	1,00*	-
		Unión estable	849/132.807	0,64	0,86	0,79; 0,94	0,88	0,80; 0,96
		Casada	423/80.814	0,52	0,70	0,63; 0,78	0,71	0,64; 0,79
	18-19	Soltera	716/90.344	0,79	1,12	1,02; 1,23	1,00*	-
		Unión estable	340/55.750	0,61	0,87	0,77; 0,98	0,77	0,67; 0,88
		Casada	79/12.699	0,62	0,88	0,70; 1,11	0,79	0,63; 1,00
	16-17	Soltera	612/66.021	0,93	1,34	1,22; 1,48	1,00*	-
		Unión estable	273/37.942	0,72	1,07	0,93; 1,22	0,79	0,68; 0,91
		Casada	2/1.838	0,11	-	-	-	-
	10-15	Soltera	285/22.601	1,26	1,91	1,67; 2,17	1,00*	-
		Unión estable	96/12.217	0,79	1,18	0,95; 1,45	0,61	0,48; 0,77
		Casada	0/321	-	-	-	-	-

Fuente: Elaboración propia a partir de datos del Instituto Nacional de Estadísticas y Censos de Ecuador ^a^Modelo multivariado ajustado por sexo del recién nacido, alfabetismo, grupo étnico, paridad (primípara, baja multípara, gran multípara), estado migratorio, región y área de residencia de la madre. Basado en modelos de regresión logística multinomial para las categorías de referencia de edad gestacional a término (37 a 42 semanas) y peso normal (≥ 2.500 g). *Valor de referencia.

Fuente: Elaboración propia a partir de datos del Instituto Nacional de Estadísticas y Censos de Ecuador.

**Figura 4:**
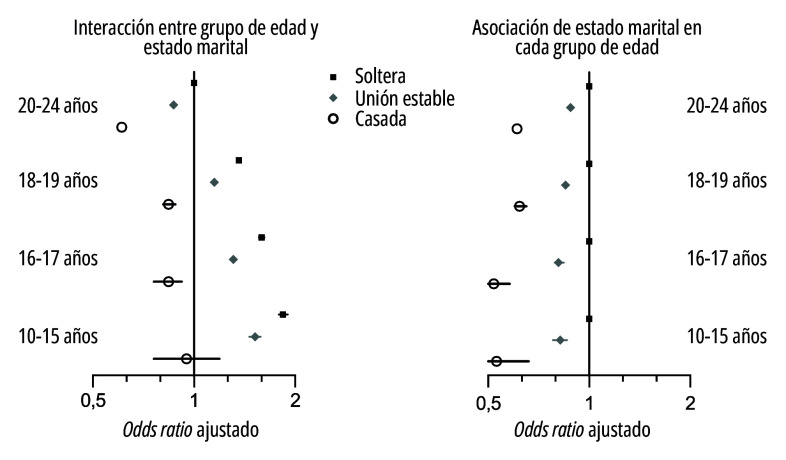
Interacción entre grupo de edad y estado marital y asociación de estado marital en cada grupo de edad, según atención prenatal y grupo de edad de la madre (n=712.902). Ecuador, 2015-2020.

Finalmente, también hubo evidencia de modificación del efecto entre el estado marital e inadecuada atención prenatal de acuerdo con la edad materna (test de interacción: valor de *p*<0,001). La inadecuada atención prenatal aumentó a medida que disminuyó la edad materna para todos los estados maritales (Tabla 4 y Figura 4), y dentro de cada grupo de edad hubo un gradiente de más alta inadecuada atención prenatal entre las mujeres solteras, seguidas de las mujeres en uniones estables y por último en las casadas (Tabla 4 y Figura 4), con diferencias ligeramente más marcadas a medida que decrece la edad materna. Con excepción de las madres de 10-15 años, las madres casadas de los otros tres grupos etarios tuvieron menor prevalencia de inadecuada atención prenatal que las madres solteras de 20-24 años. Nuevamente, las madres en uniones estables tuvieron niveles intermedios de atención prenatal inadecuada en todos los grupos etarios.

**Tabla 4:** Interacción entre grupo de edad y estado marital y asociación de estado marital en cada grupo de edad, según atención prenatal y grupo de edad de la madre (n=712.902). Ecuador, 2015-2020.

Categorías	Grupos de edad (años)	Estado marital	Eventos/nacimientos	%	Interacción entre grupo de edad y estado marital		Asociación de estado marital en cada grupo de edad	
					OR^a^	IC95%	OR^a^	IC95%
Atención prenatal	20-24	Soltera	117.561/199.548	58,91	1,00*	-	1,00*	-
		Unión estable	76.656/132.807	57,72	0,87	0,86; 0,89	0,88	0,86; 0,89
		Casada	38.331/80.814	47,43	0,61	0,60; 0,62	0,61	0,60; 0,62
	18-19	Soltera	57.177/90.344	63,22	1,36	1,34; 1,38	1,00*	-
		Unión estable	33.945/55.750	60,89	1,15	1,13; 1,17	0,85	0,83; 0,87
		Casada	6.686/12.699	52,65	0,84	0,81; 0,88	0,62	0,60; 0,65
	16-17	Soltera	43.437/66.021	65,79	1,59	1,56; 1,62	1,00*	-
		Unión estable	23.651/37.942	62,33	1,31	1,28; 1,34	0,81	0,79; 0,84
		Casada	967/1.838	52,61	0,84	0,76; 0,92	0,52	0,48; 0,58
	10-15	Soltera	15.559/22.601	68,84	1,84	1,79; 1,90	1,00*	-
		Unión estable	7.963/12.217	65,18	1,52	1,46; 1,58	0,82	0,78; 0,86
		Casada	175/321	54,52	0,95	0,76;1,19	0,53	0,42; 0,66

Fuente: Elaboración propia a partir de datos del Instituto Nacional de Estadísticas y Censos de Ecuador. Nota: Registros excluidos por dato de visitas prenatales no reportado, n = 2,179 (0,31%). ^a^Modelo mutivariado ajustado por alfabetismo, grupo étnico, paridad (primípara, baja multípara, gran multípara), residencia ecuatoriana, y región de residencia de la madre. Basado en modelos de regresión logística binaria. *Valor de referencia: Adecuada atención prenatal.

Fuente: Elaboración propia a partir de datos del instituto nacional de estadísticas y censos de ecuador

## DISCUSIÓN

### Principales hallazgos

En primer lugar, este estudio muestra una drástica reducción de casamientos legales en madres <18 años, acompañada por una reducción del 64% en uniones estables durante el periodo de estudio, mientras que la proporción de nacidos vivos de madres solteras menores de 18 años se mantuvo relativamente constante, constituyendo la mayoría. También se evidenciaron diferencias, principalmente étnicas, con mayor proporción de nacidos vivos de madres menores de 18 años en poblaciones indígenas y afroecuatorianas.

En segundo lugar, nuestros resultados confirman la asociación entre matrimonio y mejores resultados de salud entre las madres de 20-24 años de edad observada en otros estudios^8^^,^^9^^,^^10^^,^^26^, pero no proveen evidencia de una ventaja del matrimonio (y de las uniones de hecho) entre las menores de edad con respecto al bajo peso al nacer y a partos pretérmino. Vivir en pareja, sin embargo, estuvo asociado a mayor utilización de servicios de atención prenatal en todos los grupos de edad, especialmente entre las casadas legalmente, incluso, entre las menores de edad. A pesar de estas diferencias, la inadecuada atención prenatal fue alta en general en todos los subgrupos, entre el 47% y el 69%. Como ha sido observado en otros estudios, el riesgo en la salud perinatal aumenta a medida que disminuye la edad materna^26^^,^^30^^,^^31^.

### Interpretación

Si bien la proporción de nacidos vivos de mujeres menores de 18 años en Ecuador es muy alta comparada con otros países de las Américas^31^, hubo una disminución sostenida durante el periodo de estudio, del 10% en 2015 al 9% en 2018, y a menos del 8% en 2020. Esta disminución, de acuerdo con los datos del estudio, se observa en los casamientos legales (que disminuyeron del 0,62% en 2015 al 0,01% en 2020) y en las uniones informales (que disminuyeron del 4,95% en 2015 al 1,79% en 2020), pero no en los nacidos vivos de mujeres solteras menores de 18 años. La virtual desaparición de los casamientos legales entre las menores de edad parece responder a la legislación de 2015, que prohíbe el matrimonio antes de los 18 años, aunque también podría reflejar un sesgo de deseabilidad social por el cual madres menores de edad pudieron haber no reportado su verdadero estado marital por miedo a represalias o estigmatización. De cualquier manera, la nueva legislación no alcanza a las uniones estables, las cuales representan el mayor desafío, aun cuando se redujeron. Con nuestros datos, no es posible determinar si la reducción en uniones estables fue una consecuencia indirecta de la ley (sea como una verdadera disminución o como una disminución en su notificación), cuyo mensaje reprueba las uniones de menores de edad en general, o se debió a la tendencia temporal de aumento de la edad materna en general en toda la distribución de edad. 

La mayor proporción de nacidos vivos de madres <18 años entre madres indígenas y afroecuatorianas no parece estar alimentada por el casamiento infantil, sino por embarazos tempranos de madres solteras. Las altas proporciones de nacimientos en madres <18 años en las regiones de la Costa y Oriente (selva amazónica) están correlacionadas con la concentración de población afroecuatoriana e indígena en esas regiones, respectivamente^32^^,^^33^. 

El hallazgo principal de nuestro estudio es que la relación entre el estado marital y los resultados perinatales dependen de la edad materna. Observamos una ventaja del matrimonio entre las mujeres de 20 a 24 años, consistente con la literatura para partos pretérmino, bajo peso y otros resultados perinatales^8^^,^^9^^,^^10^^,^^13^^,^^14^^,^^31^, pero no entre las menores de 20 años, especialmente las menores de 18 años. Esta modificación de la asociación del matrimonio con indicadores de salud perinatal de acuerdo a la disminución de la edad materna sugiere que los mecanismos por los cuales el matrimonio influye en la salud perinatal pueden ser diferentes para mujeres adultas y niñas menores de edad. La ventaja del matrimonio adulto se suele explicar con base a dos teorías^16^, que no son mutuamente excluyentes. La primera supone un efecto causal del matrimonio, el de proporcionar un contexto propicio a conductas más saludables (por ejemplo, bajo consumo de tabaco y alcohol), que se traducen en una mejor salud. La segunda (hipótesis de selección) supone que el matrimonio no constituye una causa en sí, sino que las personas que llegan al matrimonio tienen perfiles sociodemográficos asociados con una mejor salud como, por ejemplo, mayor ingreso, riqueza, educación, y otros indicadores de posición social privilegiada. Estos mecanismos pueden no aplicar para menores de edad. El matrimonio de menores de edad puede no ser protector para las mujeres, como el matrimonio de adultos, debido a las desigualdades de género más profundas presentes en la minoría de edad, que se manifiestan como menor edad y poder con respecto al cónyuge^34^, estereotipos de género, falta de autonomía y dependencia financiera^4^^,^^5^^,^^6^^,^^7^^,^^23^. Además, los mecanismos de selección al matrimonio pueden ser diferentes entre los distintos grupos de edad y no necesariamente conferir protección a las menores, como el matrimonio presionado por miembros de la familia, impulsados por creencias religiosas, la urgencia de legitimar un embarazo no esperado, o recurrir al casamiento para escapar de la pobreza o de un entorno familiar abusivo^23^^,^^32^^,^^35^. En síntesis, lo anterior subraya la complejidad de las relaciones entre el estado marital, la edad materna y la salud perinatal y la consiguiente necesidad de emplear diversas estrategias de investigación como estudios longitudinales y cualitativos para entender las causas y consecuencias de estas relaciones.

Existe escasa información sobre el contexto y las circunstancias detrás de los nacimientos en madres menores de edad. Aunque el casamiento infantil, incluyendo uniones de hecho, es el foco de prevención en países de Asia y de África, donde los casamientos son muchas veces forzados o arreglados y la mayoría de los nacimientos ocurren dentro de estas uniones, en los países de las Américas son las mujeres solteras las que contribuyen predominantemente a los nacimientos de madres menores de edad, y a los peores resultados de salud entre las distintas situaciones maritales^31^. Aunque en las Américas las uniones forzadas no son la norma, existe la posibilidad de coerción familiar y económica, expresadas en dar una hija en matrimonio a un adulto de mayor edad y económicamente independiente o permitirlo para aliviar la pobreza familiar o bajo el supuesto de una mejora de condición para la menor^23^^,^^35^. De todas maneras, las estrategias de reducción de embarazos tempranos y sus consecuencias deben incluir a las mujeres solteras, las cuales igualmente pueden haber sido víctimas de distintos tipos de desigualdades de poder y de género y de abusos que violen su derecho al libre e informado consentimiento a mantener relaciones sexuales.

### Fortalezas y limitaciones

Entre las fortalezas del estudio se destacan el gran tamaño de los datos, de base poblacional, lo cual permitió un examen detallado de subgrupos de edad y estado marital, y la distinción entre casamientos legales y uniones informales, no disponible en los registros de nacimiento de EEUU y Canadá^9^^,^^10^. Las limitaciones son comunes a las de otros estudios que usan similares fuentes de datos^8^^,^^9^^,^^10^^,^^31^. Primero, debido a la naturaleza transversal de la recolección de datos al momento del nacimiento, el estado marital reportado pudo haber cambiado desde el momento de la concepción. Dada la ausencia de información acerca del estado marital al momento de la concepción, no es posible determinar si a la concepción preexistían o no casamientos o uniones. Esta limitación también contribuye a una subestimación de los embarazos tempranos, ya que alrededor de dos tercios de los nacimientos de madres de 18 años de edad son concebidos ocho o nueve meses antes, cuando muchas de ellas aún tienen 17 años^31^. Segundo, la información sociodemográfica es autorreportada por las madres y puede estar afectada por deseabilidad social como, por ejemplo, reportar casamiento legal en caso de uniones de hecho. En especial, las categorías preestablecidas de estado marital en el registro estadístico de nacidos vivos pueden distorsionar la peculiaridad y complejidad de los arreglos conyugales de las madres indígenas, las cuales pertenecen a diversos grupos distintivos y cuyas prácticas no pueden ser fácilmente entendidas desde una perspectiva occidental. Tercero, algunas madres pueden haber tenido más de un nacido vivo en el periodo de estudio, pero a falta de un identificador materno único en los datos, los nacidos vivos de una misma madre no pueden ser determinados. Finalmente, existe la posibilidad de confusión residual en los análisis multivariados debido a la falta de algunas variables potencialmente confusoras, como características paternas^17^, o a posibles errores de medición en algunas variables. A pesar de estas limitaciones, los resultados son relevantes para otros países de América Latina.

## CONCLUSIONES

La prohibición del matrimonio entre los menores edad contribuyó a una virtual desaparición de nacimientos de madres casadas <18 años en Ecuador, entre 2015 y 2020. A pesar de una reducción significativa de nacimientos de madres menores de edad en uniones estables, estos continúan siendo prevalentes. Nuestro estudio confirma mejores indicadores de salud reproductiva entre las madres casadas adultas, pero no necesariamente entre las casadas menores de edad. A diferencia de lo que ocurre en países de Asia y África, en Ecuador las madres solteras aportan la mayor cantidad de los partos en madres menores de edad, con resultados de salud más bajos que en las que están en pareja, razón por la cual las estrategias de prevención de nacimientos tempranos deben incluir a las mujeres solteras menores de edad y fortalecer la autonomía de las adolescentes en materia de derechos sexuales y reproductivos y de formación de familia.
