# Supplementary material for: Casamiento infantil y salud perinatal en Ecuador, 2015-2020
Source: Salud Colect. 2023 Apr 13;19:e4325. [Article in Spanish] doi: 10.18294/sc.2023.4325 (PMC11930324; doi:10.18294/sc.2023.4325)
Supplement: Translation

# Translation: Child marriage and perinatal health in Ecuador, 2015-2020

**Authors:** Dorian Ospina Galeano, Fadya Asia Orozco, Marcelo Luis Urquia

**Affiliations:** 1 Gerente de Sistemas de Información en Salud. Estudiante, Community Health Sciences (MSc), Rady Faculty of Health Sciences, University of Manitoba, Winnipeg, Manitoba, Canadá. ospinagd@myumanitoba.ca; 2 Doctora en Salud Pública. Docente Titular, Escuela de Salud Pública, Universidad San Francisco de Quito, Quito, Ecuador. forozco@usfq.edu.ec; 3 PhD in Epidemiology. Associate Professor, Department of Community Health Sciences, Rady Faculty of Health Sciences, University of Manitoba, Winnipeg, Manitoba, Canadá. marcelo.urquia@umanitoba.ca

**Keywords:** Maternal Age, Live Birth, Marital Status, Birth Weight, Premature Birth, Ecuador

## Abstract

This study sought to estimate the prevalence and distribution of newborns to mothers under age 18 in Ecuador and the association between perinatal indicators and maternal marital status. Newborn records obtained from Ecuador’s Instituto Nacional de Estadísticas y Censos (INEC) between 2015 and 2020 were used to assess the joint association between maternal age groups (10-15, 16-17, 18-19, and 20-24 years) and marital status (married, common-law, and single) with low birthweight, preterm birth, and inadequate prenatal care. The prevalence of newborns to mothers under age 18 was 9.3% overall, but declined over the study period, drastically among married mothers. The association between marital status and perinatal indicators depended on maternal age. The more favorable outcomes observed among married mothers aged 20-24 years (compared to their single counterparts) weaken or disappear among mothers under age 18. Mothers in stable unions exhibited outcomes in between those of married and single mothers.

## INTRODUCTION

Reducing adolescent pregnancies and child marriage are considered key objectives in preventing early pregnancy and poor reproductive health outcomes among adolescents.^1^

Child marriage, defined as a legally formalized or informal (“common-law”) union before the age of 18, is increasingly recognized by various organizations and governments worldwide as a threat to human rights, particularly those of girls.^2^^,^^3^^,^^4^ Marriage before the age of 18 is more common for girls than it is for boys, and girls often marry at a younger age, reflecting gender inequalities that can have a negative impact on women’s health, education, and autonomous development throughout the course of their lives.^5^

Each year, approximately 12 million girls are married before the age of 18.^5^ Although child marriage is more prevalent in Sub-Saharan Africa and South Asia,^6^^,^^7^ it is a global phenomenon, with a high prevalence in Latin American countries such as Brazil,^8^ and even in countries such as Canada and the United States.^9^^,^^10^^,^^11^^,^^12^

Married women generally have better perinatal health outcomes than unmarried women,^13^^,^^14^^,^^15^ whereas those in common-law unions exhibit intermediate outcomes.^14^^,^^15^ The advantages of adult marriage are thought to stem from a number of factors: the positive influence of marriage as a social institution (for example, this hypothesis holds that marriage contributes to the adoption and maintenance of healthier attitudes and behaviors, as well as the prevention of risk behaviors); that individuals who choose to marry are generally healthier (for example, this hypothesis states that it is not marriage itself but rather that individuals who eventually choose to marry have more privileged socioeconomic backgrounds and are therefore healthier than those who do not marry); or, a combination of these factors.^16^^,^^17^ Regardless of the mechanisms involved, the issue of whether or not the health benefits associated with adult marriage also extend to girls under age 18 has not been well studied.

Most of the literature on child marriage and its social and health consequences comes from studies carried out in Asian and African countries, where the majority of early pregnancies occur in the context of arranged marriages.^5^ These studies link child marriage with lower levels of schooling among girls, limited autonomy, domestic violence, unwanted pregnancies, higher fertility, and poorer reproductive health outcomes in comparison to women who marry in adulthood.^18^^,^^19^^,^^20^^,^^21^^,^^22^ However, these associations cannot be easily generalized to the Latin American context, where most pregnancies occur outside of formal marriages, childbirth outside of marriage is increasingly accepted, and the majority of marriages and common-law unions are purportedly consensual.^23^

In Ecuador, 18 is the legal age of majority, defined as the minimum age at which citizens are considered capable of making responsible decisions and can freely exercise rights and responsibilities in society. In 2015, Ecuador modified its Civil Code to prohibit marriage for minors of both sexes under 18 years of age. Up to that point, the minimum marriage age was 14 for boys and 12 for girls.^24^ As of the 2020s, child marriage is outlawed in only nine Latin American and two Caribbean countries.^25^

Although many quantitative perinatal studies group pregnant women under 20 years of age into a single category in order to overcome sample size limitations or to facilitate comparisons with other age groups, this group exhibits high levels of heterogeneity in terms of risk, reflecting the influence of various factors such as nutrition, menarche, and gynecologic age.^26^ Child marriage and minor status are sociocultural factors that further highlight the importance of distinguishing age subgroups among pregnant mothers under 20 in reproductive health studies in order to examine the spectrum of risk levels among adolescents and minors in greater detail, particularly with respect to the intersection between maternal age and marital status. 

To contribute to the knowledge on the complex relations between marital status, maternal age, and reproductive health indicators, this study analyzes all live births in Ecuador since 2015, the year in which child marriage was prohibited. This study aims to estimate the prevalence and distribution of live births to mothers under age 18 in Ecuador and the association between perinatal indicators and marital status of mothers who had live births in the following age groups: 10-15, 16-17, 18-19, and 20-24 years of age. This study provides useful information for understanding the specificities related to marital status among minors, adolescents, and young adults, as well as associations with reproductive health indicators.

## MATERIALS AND METHODS

A population-based cross-sectional study was carried out based on live birth records from 2015-2020 obtained from Ecuador’s National Institute of Statistics and Census (INEC). As of 2015, Ecuador has implemented online electronic birth certificates through the National Vital Data Registration System (REVIT), which gradually grew to include 88 healthcare establishments in 2015, 569 in 2017, and 606 in 2020.^27^ Physical certificates of live birth have continued to be used for non-institutional births and in institutions that have not yet been incorporated into the REVIT; these are downloaded by the institution, completed, and returned to the INEC for processing.^27^ Both online electronic forms and physical certificates are compiled and sent to INEC’s Directorate of Administrative Records (DIRAD) on a monthly basis, where quality evaluation and information processing are performed. Inconsistencies in the data are resolved with other sources of information (Civil Registry Office and healthcare establishments) prior to being entered into INEC databases.^27^

### Study population

A total of 1,826,456 live births were registered during the study period. Of these, 116,468 (6.4%) cases were excluded because they occurred before 2015, 51,609 (3.0%) because they were registered after March 31 of the year following the birth, and 3,277 (0.2%) because of missing data on birth year. An additional 81 (0.004%) cases were excluded that corresponded to live births to women over age 49, and 25,314 (1.5%) because of missing data on the mother’s age. This left a total of 1,629,707 live births to mothers aged 10 to 49 for the estimation of the proportion of mothers under age 18 among all mothers of reproductive age. 

To evaluate the relationship between low birth weight, preterm birth, and prenatal care according to the marital status of mothers who were minors, adolescents, and young adults, the following cases were excluded: 877,926 live births to mothers aged 25 and over; 7,558 cases of multiple births; 5,544 that had missing data on ethnicity or maternal marital status; 20,823 that had missing data on birth weight or gestational age; 860 cases in which birth weight exceeded four standard deviations from the sex- and gestational age-specific mean birth weight; and 4094 cases of live births to mothers who were divorced, separated, or widowed, or who had missing data on place of residence. Some cases met more than one of the exclusion criteria. Therefore, the study population for multivariate analysis of low birth weight and preterm births included 712,902 live births to mothers from 10 to 24 years of age. For the analysis of inadequate prenatal care, 710,723 cases were considered after excluding cases with no data on prenatal care.

### Measures

Marital status of mothers was categorized as follows: legally married, stable union, single, widowed/separated/divorced, and unreported. For descriptive analyses intended to determine the prevalence of child marriage, live births to widowed/separated/divorced mothers were grouped with married mothers since they had been previously married. However, due to the low number of live births to widowed/separated/divorced women and those with missing data on marital status, these groups were not included as comparison groups in multivariate analyses. 

Maternal age was grouped in the following categories: 10-15, 16-17, 18-19, 20-24, and 25-49 years of age. The 25-49 age group was not included in multivariate analyses given that the focus of this study was on minors, adolescents, and young adult mothers. 

Low birth weight (<2,500 g) was subdivided into very low birth weight (<1,500 g) and moderately low birth weight (1,500-2,499 g), with normal birth weight of ≥2,500 g as the reference group. Preterm birth (<37 weeks of gestation) was subdivided into very preterm (24-31 weeks) and moderately preterm (32-36 weeks), with full-term births (37 or more weeks) as the reference group.

Prenatal care was categorized as adequate or inadequate, based on the Revised Graduated Prenatal Care Utilization Index (Revised-GINDEX),^28^ which combines information on number of prenatal care visits and gestational age.

### Statistical analysis

For descriptive analyses intended to estimate the prevalence of mothers under 18 years of age by marital status and sociodemographic characteristics, proportions expressed as percentages were calculated, with all live births to mothers aged 10-49 as the denominator. 

In order to evaluate the associations between maternal marital status and low birth weight, preterm birth, and prenatal care, only mothers aged 10 to 24 years were considered. Multinomial logistic regression was used to model the two levels of low birth weight (very low and moderately low) and of preterm birth (very preterm and moderately preterm), whereas binomial logistic regression was used for inadequate prenatal care (yes vs. no). To assess the interaction between age groups and maternal marital status, an interaction term for these variables was included in the models. Statistical significance of the interaction was measured with a likelihood-ratio test, which compares the model “Ln(Odds) = marital status + age group + marital status * age group” with the simpler model “Ln(Odds) = marital status + age group” with the null hypothesis that the more complex model is not more informative than the simpler model. In order to show the joint influence of marital status and maternal age on the dependent variables, the results of each model are presented in two ways: i) taking single women aged 20-24 as the only reference category; and ii) taking single women as the reference category within each age group, equivalent to a stratified analysis.^29^

Models were adjusted for sex of newborn (except for prenatal care), literacy (yes/no), primiparity (yes/no), immigrant status (yes/no), ethnic group (Indigenous/Afro-Ecuadorian/mestiza/white/other), area of residence (rural/urban), and region of residence (Coastal/Eastern/Mountain/Islander/other) of the mother. 

In order to avoid unstable and imprecise estimations, we only reported associations based on ten or more cases in the subgroups defined by the intersection between marital status and maternal age group.

Data manipulation and statistical analysis were performed using SAS and R statistical programs. Graphics were created in Excel and Prisma. Ecuador’s live birth database is public domain and therefore no Ethical Review Board approval is needed for its use. 

## RESULTS

There were 1,629,707 live births to women aged 10-49 in Ecuador from 2015 to 2020 (see Table 1a and Table 1b). Of these, 147,936 (9.1%) were to mothers under age 18, of which 36,934 (25%) were to mothers aged 10-15. The proportion of legally married women increased with maternal age, although it was very low among those aged 18 and under. Nonetheless, one third of those who had children between 10-15 and 16-17 years of age were in stable unions.

**Table 1a:** Distribution of live births according to characteristics of the mother, by maternal age group. Ecuador, 2015-2020.

Characteristics of the mother	Maternal age in years											
	10-15		16-17		18-19		20-24		25-49		Total	
	n	%	n	%	n	%	n	%	n	%	n	%
Total	36,934	2.3	111,002	6.8	167,157	10.3	436,688	26.8	877,926	53.9	1,629,707	100.0
Marital status												
Married	349	0.9	2,026	1.8	13,711	8.2	85,914	19.7	376,707	42.9	478,707	29.4
table union	12,719	34.4	39,437	35.5	58,059	34.7	138,380	31.7	198,929	22.7	447,524	27.5
Single	23,607	63.9	68,795	62.0	94,136	56.3	207,681	47.6	270,040	30.8	664,259	40.8
Separated, divorced or widowed	72	0.2	257	0.2	428	0.3	2,555	0.6	28,133	3.2	31,445	1.9
Not reported	187	0.5	487	0.4	823	0.5	2,158	0.5	4,117	0.5	7,772	0.5
Primiparity												
Yes	35,824	97.0	99,793	89.9	128,363	76.8	209,958	48.1	172,152	19.6	646,090	39.6
No	1,110	3.0	11,209	10.1	38,794	23.2	226,730	51.9	705,774	80.4	983,617	60.4
Literacy												
Yes	36,609	99.1	110,367	99.4	166,363	99.5	434,230	99.4	869,446	99.0	1,617,015	99.2
No	199	0.5	330	0.3	35	0.2	1,273	0.3	5,969	0.7	8,128	0.5
Not reported	126	0.3	305	0.3	437	0.3	1,185	0.3	2,511	0.3	4,564	0.3
Ethnic group												
Indigenous	2,696	7.3	8,294	7.5	11,882	7.1	26,581	6.1	49,014	5.6	98,467	6.0
Afro-Ecuatorian	1,230	3.3	3,037	2,7	4,251	2.5	11,041	2.5	20,145	2.3	39,704	2.4
Mestiza	32,509	88.0	98,430	88.7	148,979	89.1	392,821	90.0	792,846	90.3	1,465,585	89.9
White	240	0.6	634	0.6	974	0.6	2,981	0.7	8,524	1.0	13,353	0.8
Not reported	259	0.7	607	0.5	1,071	0.6	3,264	0.7	7,397	0.8	12,598	0.8
Area of residence												
Rural	9,654	26.1	28,327	25.5	40,583	24.3	95,776	21.9	183,798	20.9	358,138	22.0
Urban	27,280	73.9	82,675	74.5	126,574	75.7	340,912	78.1	694,128	79.1	1,271,569	78.0
Region of residence												
Coastal	23,408	63.4	65,599	59.1	96,607	57.8	249,903	57.2	453,396	51.6	888,913	54.5
Mountain	9,693	26.2	36,163	32.6	58,568	35.0	158,994	36.4	371,588	42.3	635,006	39.0
Eastern	3,793	10.3	9,088	8.2	11,747	7.0	27,064	6.2	51,086	5.8	102,778	6.3
Islander	24	0.1	93	0.1	168	0.1	572	0.1	1,504	0.2	2,361	0.1
Other	16	<0.1	59	<0.1	67	<0.1	155	<0.1	352	<0.1	649	<0.1
Ecuadorian citizenship												
Yes	36,304	98.3	109,088	98.3	162,955	97.5	422,387	96.7	848,929	96.7	1,579,663	96.9
No	615	1.7	1,883	1.7	4,156	2.5	14,178	3.2	28,692	3.3	49,524	3.0
No reported	15	<0.1	31	<0.1	46	<0.1	123	<0.1	305	<0.1	520	<0.1

Source: Own elaboration based on data from Ecuador’s National Institute of Statistics and Census.

**Table 1b:** Distribution of live births according to characteristics of the birth and the newborn, by maternal age group. Ecuador, 2015-2020

Characteristics of the birth and the newborn	Maternal age in years											
	10-15		16-17		18-19		20-24		25-49		Total	
	n	%	n	%	n	%	n	%	n	%	n	%
Total	36,934	2.3	111,002	6.8	167,157	10.3	436,688	26.8	877,926	53.9	1,629,707	100.0
Birth year												
2015-2016	13,345	36.1	38,438	34.6	56,300	33.7	141,010	32.3	277,008	31.6	526,101	32.3
2017-2018	13,088	35.4	39,665	35.7	57,866	34.6	153,545	35.2	304,245	34.7	568,409	34.9
2019-2020	10,501	28.4	32,899	29.6	52,991	31.7	142,133	32.5	296,673	33.8	535,197	32.8
Sex												
Male	18,676	50.6	57,051	51.4	85,571	51.2	223,920	51.3	447,508	51.0	832,726	51.1
Female	18,258	49,4	53,951	48.6	81,586	48.8	212,768	48.7	430,418	49.0	796,981	48.9
Birth weight												
Very low (<1,500 g)	494	1.3	1,205	1.1	1,649	1.0	4,077	0.9	10,056	1.1	17,481	1.1
Moderately low (1,500-2,499 g)	4,006	10.8	10,652	9.6	14,419	8.6	33,236	7.6	66,901	7.6	129,214	7.9
Normal (> 2,499 g)	31,571	85.5	96,655	87.1	147,348	88.1	390,563	89.4	784,534	89.4	1,450,671	89.0
Not reported	863	2.3	2,490	2.2	3,741	2.2	8,812	2.0	16,435	1.9	32,341	2.0
Gestational age												
Very preterm (24-31 weeks)	465	1.3	1,092	1.0	1,472	0.9	3,564	0.8	9,113	1.0	15,706	1.0
Moderately preterm (32-36 weeks)	2,705	7.3	6,727	6.1	9,121	5.5	22,719	5.2	56,012	6.4	97,284	6.0
Full-term (37-42 weeks)	32,708	88.6	100,095	90.2	15,191	90.9	399,091	91.4	790,618	90.1	1,474,429	90.5
Not reported	1,056	2.9	3,088	2.8	4,647	2.8	11,314	2.6	22,183	2.5	42,288	2.6
Prenatal care												
No visits	876	2.4	1,851	1.7	2,685	1.6	5,595	1.3	8,135	0.9	19,142	1.2
Inadequate	23,146	62.7	67,077	60.4	96,496	57.7	231,280	53.0	372,653	42.4	790,652	48.5
Adequate	11,712	31.7	38,604	34.8	62,794	37.6	186,957	42.8	471,330	53.7	771,397	47.3
Not reported	1,200	3.2	3,470	3.1	5,182	3.1	12,856	2.9	25,808	2.9	48,516	3.0
Multiplicity												
Single birth	36,678	99.3	110,186	99.3	165,621	99.1	431,738	98.9	861,661	98.1	1,605,884	98.5
Multiple birth	256	0.7	816	0.7	1,536	0.9	4,950	1.1	16,265	1.9	23,823	1.5
Type of delivery												
Normal	24,633	66.7	75,780	68.3	108,444	64.9	251,638	57.6	398,397	45.4	858,892	52.7
Cesarean	12,242	33.1	35,075	31.6	58,450	35.0	184,432	42.2	478,392	54.5	768,591	47.2
Not reported	59	0.2	147	0.1	263	0.2	618	0.1	1,137	0.1	2,224	0.1
Type of healthcare provider at delivery												
Midwife or traditional healer	969	2.6	2,796	2.5	4,217	2.5	9,972	2.3	18,168	2.1	36,122	2.2
Healthcare professional	35,965	97.4	108,206	97.5	162,940	97.5	426,716	97.7	859,758	97.9	1,593,585	97.8
Place of birth												
Private healthcare institution	4,408	11.9	14,920	13.4	26,385	15.8	81,903	18.8	227,806	25.9	355,422	21.8
Public healthcare institution	31,442	85.1	92,922	83.7	135,923	81.3	343,360	78.6	628,951	71.6	1,232,598	75.6
Home birth or other	1,084	2.9	3,160	2.8	4,849	2.9	11,425	2.6	21,169	2.4	41,687	2.6

Source: Own elaboration based on data from Ecuador’s National Institute of Statistics and Census.

The proportion of mothers under age 18 decreased from 10.0% in 2015 to 7.8% in 2020, mainly due to a reduction in the number of women that were married and in stable unions (Figure 1). The decrease in married mothers was nearly total, from 0.62% in 2010 to 0.01% in 2020. Live births to mothers in stable unions also fell from 4.95% in 2015 to 1.79% in 2020 - a 64% reduction - whereas the proportion of live births to single mothers remained relatively stable. The prevalence of live births to mothers under age 18 was higher than the national average in the Coastal and Eastern regions, as well as among mothers of Indigenous or Afro-Ecuadorian background, with the highest proportions of single mothers. 

**Figure 1:** Percentage of live births to mothers under age 18, by marital status and sociodemographic characteristics (n=712,902). Ecuador, 2015-2020.

Source: Own elaboration based on data from Ecuador’s National Institute of Statistics and Census.

In multivariate analyses of low birth weight (Table 2 and Figure 2), it was possible to observe a clear and gradual increase in the proportion of live births with moderately low and very low birth weight as maternal age decreased (Figure 2). The association between marital status and birth weight was slightly modified by maternal age (interaction test p-value of 0.06). Within each age group (Figure 2), married mothers in the 18-19 and 20-24 age groups had lower chances (odds ratios) of moderately low and very low birth weight than single mothers, but no significant differences in terms of moderately low birth weight were observed in the 10-15 and 16-17 age groups. Due to the small number of live births with very low birth weight to married mothers aged 10-15 and 16-17, odds ratios were not estimated. Live births to mothers in stable unions represented an intermediate situation between married and single mothers.

**Table 2:** Interaction between age group and marital status and association for marital status within each age group, by birth weight and maternal age group (n=712,902). Ecuador, 2015-2020.

Categories	Age groups (years)	Marital status	Events/births	%	Interaction between age group and marital status		Association for marital status within age group	
					OR^a^	CI95%	OR^a^	CI95%
Moderately low birth weight (1,500 – 2,499 g)	20-24	Single	14,914/ 199,548	7.47	1.00*	-	1.00*	-
		Stable union	9,189/ 132,807	6.92	0.98	0.95; 1.01	0.98	0.96; 1.01
		Married	5,514/ 80,814	6.82	0.89	0.86; 0.91	0.89	0.86; 0.92
	18-19	Single	7,829/ 90,344	8.67	1.13	1.10; 1.17	1.00*	-
		Stable union	4,349/ 55,750	7.60	1.06	1.04; 1.12	0.95	0.91; 0.99
		Married	1,048/ 12,699	8.25	1.04	0.97; 1.11	0.92	0.80; 0.98
	16-17	Single	6,378/ 66,021	9.66	1.27	1.23; 1.31	1.00*	-
		Stable union	3,422/ 37,642	9.02	1.26	1.23; 1.31	0.99	0.91; 1.04
		Married	168/ 1,838	9.14	1.28	1.00; 1.38	0.92	0.78; 1.08
	10-15	Single	2,542/ 22,601	11.25	1.53	1.46; 1.60	1.00*	-
		Stable union	1,197/ 12,217	9.80	1.39	1.30; 1.48	0.90	0.84; 0.97
		Married	37/ 321	11.53	1.54	1.09; 2.17	1.01	0.71; 1.42
Very low birth weight (< 1,500 g)	20-24	Single	1,540/ 199,548	0.77	1.00*	-	1.00*	-
		Stable union	953/ 132,807	0.72	0.93	0.86; 1.01	0.94	0.86; 1.02
		Married	468/ 80,814	0.56	0.75	0.68; 0.84	0.78	0.69; 0.84
	18-19	Single	726/ 90,344	0.80	1.06	0.97; 1.16	1.00*	-
		Stable union	416/ 55,750	0.75	0.99	0.89; 1.10	0.93	0.82; 1.05
		Married	77/12,699	0.61	0.81	0.64; 1.02	0.76	0.60; 0.96
	16-17	Single	622/66,021	0.94	1.26	1.14; 1.39	1.00*	-
		Stable union	283/37,942	0.75	1.00	0.88; 1.14	0.78	0.67; 0.9
		Married	7/1,838	0.38	-	-	-	-
	10-15	Single	257/22,601	1.14	1.56	1.36; 1.79	1.00*	-
		Stable union	114/12,217	0.93	1.27	1.05; 1.55	0.83	0.66; 1.04
		Married	0/321	-	-	-	-	-

Source: Own elaboration based on Ecuador’s National Institute of Statistics and Census. ^a^Multivariate model adjusted for sex of newborn, literacy, ethnic group, parity (primiparity, low multiparity, grand multiparity), migration status, region and place of residence of the mother. Based on multinomial logistic regression models with reference categories of full-term gestational age (37 to 42 weeks) and normal birth weight (≥ 2,500 g). *Reference category.

**Figure 2:** Interaction between age group and marital status and association for marital status within each age group, by birth weight and maternal age group (n=712,902). Ecuador, 2015-2020.

Source: Own elaboration based on Ecuador’s National Institute of Statistics and Census.

A similar gradient of increasing preterm births with lower maternal age could be observed, both for 24-31 weeks and 32-36 weeks of gestational age (Table 3 and Figure 3). The association between marital status and preterm births was modified by maternal age (interaction test p-value of <0.001). In the 20-24 age group, preterm births were less frequent among married women, followed by women in stable unions, and were highest among single women (Table 3 and Figure 3). This pattern was even more pronounced for 24-31 weeks of gestational age. However, this pattern was not observed in mothers aged 18-19 or in mothers under age 18. Mothers in stable unions had a lower frequency of very preterm births than single mothers in all age groups. Due to the virtual absence of very preterm births among married women mothers under age 18, odds ratios could not be calculated.

**Table 3:** Interaction between age group and marital status and association for marital status within each age group, by preterm birth and maternal age group (n=712,902). Ecuador, 2015-2020.

Categories	Age groups (years)	Marital status	Events/births	%	Interaction between age group and marital status		Association for marital status within age group	
					OR^a^	CI95%	OR^a^	CI95%
Moderately preterm birth (32-36 weeks)	20-24	Single	9,929/199,548	4.98	1.00*	-	1.00*	-
		Stable union	6,233/132,807	4.69	0.98	0.95; 1.01	0.98	0.95; 1.02
		Married	3,814/80,814	4.72	0.92	0.88; 0.95	0.92	0.89; 0.96
	18-19	Single	4,887/90,344	5.41	1.14	1.10; 1.18	1.00*	-
		Stable union	2,730/55,750	4.90	1.09	1.04; 1.14	0.94	0.90; 0.99
		Married	620/12,699	4.88	1.00	0.92; 1.09	0.88	0.81; 0.96
	16-17	Single	4,015/66,021	6.08	1.32	1.27; 1.37	1.00*	-
		Stable union	2,093/37,942	5.52	1.28	1.22; 1.34	0.97	0.92; 1.03
		Married	107/1,838	5.82	1.24	1.02; 1.51	0.91	0.75; 1.11
	10-15	Single	1,767/22,601	7.82	1.80	1.70; 1.89	1.00*	-
		Stable union	731/12,217	5.98	1.44	1.33; 1.56	0.82	0.75; 0.90
		Married	21/321	6.54	1.46	0.93; 2.28	0.81	0.52; 1.27
Very preterm birth (24-31 weeks)	20-24	Single	1,479/199,548	0.74	1.00*	-	1.00*	-
		Stable union	849/132,807	0.64	0.86	0.79; 0.94	0.88	0.80; 0.96
		Married	423/80,814	0.52	0.70	0.63; 0.78	0.71	0.64; 0.79
	18-19	Single	716/90,344	0.79	1.12	1.02; 1.23	1.00*	-
		Stable union	340/55,750	0.61	0.87	0.77; 0.98	0.77	0.67; 0.88
		Married	79/12,699	0.62	0.88	0.70; 1.11	0.79	0.63; 1.00
	16-17	Single	612/66,021	0.93	1.34	1.22; 1.48	1.00*	-
		Stable union	273/37,942	0.72	1.07	0.93; 1.22	0.79	0.68; 0.91
		Married	2/1,838	0.11	-	-	-	-
	10-15	Single	285/22,601	1.26	1.91	1.67; 2.17	1.00*	-
		Stable union	96/12,217	0.79	1.18	0.95; 1.45	0.61	0.48; 0.77
		Married	0/321	-	-	-	-	-

Source: Own elaboration based on Ecuador’s National Institute of Statistics and Census. ^a^Multivariate model adjusted for sex of newborn, literacy, ethnic group, parity (primiparity, low multiparity, grand multiparity), migration status, region and place of residence of the mother. Based on multinomial logistic regression models with reference categories of full-term gestational age (37 to 42 weeks) and normal birth weight (≥ 2,500 g). *Reference category.

**Figure 3:** Interaction between age group and marital status and association for marital status within each age group, by preterm birth and maternal age group (n=712,902). Ecuador, 2015-2020.

Source: Own elaboration based on Ecuador’s National Institute of Statistics and Census.

Lastly, there was also evidence of a modification of the association between marital status and inadequate prenatal care according to maternal age (interaction test p-value of <0.001). Inadequate prenatal care increased with lower maternal age for all marital statuses (Table 4 and Figure 4), and within each age group there was a range of higher inadequate prenatal care among single mothers, followed by mothers in stable unions, and lastly married mothers (Table 4 and Figure 4), with slightly more pronounced differences as maternal age decreased. With the exception of mothers aged 10-15, married mothers in the other three age groups had a lower prevalence of inadequate prenatal care than single women 20-24 years of age. Again, mothers in stable unions had intermediate levels of prenatal care in all age groups.

**Table 4:** Interaction between age group and marital status and association for marital status within each age group, by prenatal care and maternal age group (n=712,902). Ecuador, 2015-2020.

Categories	Age groups (years)	Marital status	Events/births	%	Interaction between age group and marital status		Association for marital status within age group	
					OR^a^	CI95%	OR^a^	CI95%
Prenatal care	20-24	Single	117.561/199.548	58.91	1.00*	-	1.00*	-
		Stable union	76.656/132.807	57.72	0.87	0.86; 0.89	0.88	0.86; 0.89
		Married	38.331/80.814	47.43	0.61	0.60; 0.62	0.61	0.60; 0.62
	18-19	Single	57.177/90.344	63.22	1.36	1.34; 1.38	1.00*	-
		Stable union	33.945/55.750	60.89	1.15	1.13; 1.17	0.85	0.83; 0.87
		Married	6.686/12.699	52.65	0.84	0.81; 0.88	0.62	0.60; 0.65
	16-17	Single	43.437/66.021	65.79	1.59	1.56; 1.62	1.00*	-
		Stable union	23.651/37.942	62.33	1.31	1.28; 1.34	0.81	0.79; 0.84
		Married	967/1.838	52.61	0.84	0.76; 0.92	0.52	0.48; 0.58
	10-15	Single	15.559/22.601	68.84	1.84	1.79; 1.90	1.00*	-
		Stable union	7.963/12.217	65.18	1.52	1.46; 1.58	0.82	0.78; 0.86
		Married	175/321	54.52	0.95	0.76; 1.19	0.53	0.42; 0.66

Source: Own elaboration based on Ecuador’s National Institute of Statistics and Census Note: Excluded cases due to missing data on prenatal visits, n = 2,179 (0.31%). ^a^Multivariate model adjusted for literacy, ethnic group, parity (primiparity, low multiparity, grand multiparity), Ecuadorian residence, region and place of residence of the mother. Based on binary logistic regression models. *Reference category: Adequate prenatal care.

**Figure 4:** Interaction between age group and marital status and association for marital status within each age group, by birth weight and maternal age group (n=712,902). Ecuador, 2015-2020.

Source: Own elaboration based on Ecuador’s National Institute of Statistics and Census.

## DISCUSSION

### Main findings

In the first place, this study shows that there was a drastic reduction in legal marriages of mothers under age 18 over the course of the study period, along with a 64% reduction in the number of stable unions, while the proportion of live births to single mothers under age 18 remained relatively stable, constituting the majority. Differences were also observed with respect to ethnicity, with higher proportions of live births to mothers under age 18 in Indigenous and Afro-Ecuadorian populations.

Secondly, our results confirm the association between marital status and better health outcomes among mothers aged 20-24 that has been observed in other studies,^8^^,^^9^^,^^10^^,^^26^ but they do not provide evidence of a marriage advantage (or that of stable unions) among minors with respect to low birth weight and preterm birth. Being in a stable union, however, was associated with higher utilization of prenatal care services in all age groups, especially among legally married women, even among minors. Despite these differences, inadequate prenatal care was generally high in all subgroups, ranging from 47% to 69%. As other studies have pointed out, prenatal health risks are higher with decreasing maternal age.^26^^,^^30^^,^^31^

### Interpretation

Although the proportion of live births to minors is very high in Ecuador compared to other countries in the Americas,^31^ a steady decrease could be observed during the study period, from 10% in 2015 to 9% in 2018, to less than 8% in 2020. According to the data analyzed in this study, this decline could be observed in women who were legally married (decreasing from 0.62% in 2015 to 0.01% in 2020) and in stable unions (decreasing from 4.95% in 2015 to 1.79% in 2020), but not in live births to single mothers under age 18. The virtual disappearance of legal marriages among minors seems to be a consequence of the 2015 legislation prohibiting marriage before the age of 18, although it could also reflect a social desirability bias, whereby minor mothers may not have reported their true marital status out of fear of social stigmatization or other repercussions. In any case, this recent legislation does not contemplate stable unions, which currently represent one of the most significant challenges despite a reduction in number. With our data, it is not possible to determine whether the reduction in stable unions was an indirect consequence of the legislation - which in principle condemns all types of unions among minors - either as a real decrease or merely a decline in reporting, or if it was due to the trend toward increasing maternal age across the entire age distribution.

The higher proportion of live births to mothers under age 18 among Indigenous and Afro-Ecuadorian mothers does not seem to be caused by child marriage, but rather by early pregnancies among single mothers. The high proportions of births among mothers under age 18 in the Coastal and Eastern (Amazon) regions are correlated with the higher concentration of Afro-Ecuadorian and Indigenous population in these regions respectively.^32^^,^^33^

The main finding of our study is that the relationship between marital status and perinatal outcomes depends on maternal age. We observed a marriage advantage among women aged 20 to 24, which was consistent with the literature on preterm birth, low birth weight, and other perinatal outcomes,^8^^,^^9^^,^^10^^,^^13^^,^^14^^,^^31^ but not among women under age 20, in particular those under age 18. This modification of the association of marriage with perinatal health indicators according to lower maternal age suggests that the mechanisms through which marriage influences perinatal health may be different for adult women than for minors. The marriage advantage for adults is usually understood in terms of two theories,^16^ which are not mutually exclusive. The first assigns a causal effect to marriage, in that it provides a context which promotes healthier behaviors (for example, lower levels of smoking and alcohol consumption) which translate to better health outcomes. The second (called the marriage selection hypothesis) assumes that marriage itself is not a cause, but rather that individuals who eventually choose to marry have sociodemographic characteristics associated with better health, such as higher income, wealth, education, and other indicators of more privileged socioeconomic backgrounds. These mechanisms may not be the same for minors. Marriage among minors may not be a protective factor for minors in the same way it may be for adult women due to the deeper gender inequalities that are present for minors, which manifest themselves in the lower age and power in comparison to their spouse,^34^ gender stereotypes, lack of autonomy, and financial dependence.^4^^,^^5^^,^^6^^,^^7^^,^^23^ Furthermore, marriage selection mechanisms may differ among age groups - and may not necessarily confer the same protective aspects to minors - including the pressure to marry by family members (driven by religious beliefs), the urgency to legitimize an unintended pregnancy, or the idea that marriage is a way to escape poverty or abuse in the family of origin.^23^^,^^32^^,^^35^ To summarize, these aspects point to the complexity of the relationship between marital status, maternal age, and perinatal health, and the consequent need to employ diverse research strategies such as longitudinal and qualitative studies in order to understand the causes and consequences of these relationships.

There is little information regarding the context and circumstances surrounding births to mothers who are minors. Although child marriage (including common-law unions) is the focus of the prevention strategies in Asia and Africa - where forced or arranged marriages are more common and the majority of births to minors occur under these circumstances - in the Americas, single mothers account for the majority of cases of births to minors and the worse health outcomes among different marital arrangements.^31^ Although forced marriages are not the norm in the Americas, the possibility of family and economic coercion does exist, for example when young daughters are married off to economically independent adult men as a means to alleviate family poverty under the guise of providing better economic conditions for the minor.^23^^,^^35^ In any case, strategies for the reduction of early pregnancies and their consequences should contemplate single women, who also may also have been victims of different types of gender and power inequalities or forms of abuse that violate their right to free and informed consent to engage in sexual relations.

### Strengths and limitations

Among the strengths of this study, the large volume of population-based data stands out, which allowed for a detailed analysis of different age and marital status subgroups, along with the ability to distinguish between legal marriages and common-law unions, something that is not possible when analyzing birth records in the USA and Canada.^9^^,^^10^ The limitations are comparable to other studies that use similar data sources.^8^^,^^9^^,^^10^^,^^31^ First, due to the cross-sectional nature of data collection at the time of birth, reported marital status may have changed since the time of conception. Since data is not collected on marital status at the time of conception, it is not possible to determine if a preexisting marriage or union existed when the child was conceived. This limitation also contributes to an underestimation of early pregnancies, given that around two thirds of births to 18-year-old mothers were conceived eight or nine months prior, when many of them were still 17 years of age.^31^ Second, sociodemographic information is self-reported by mothers and may be affected by social desirability biases, for example reporting legal marriage in the case of common-law unions. In particular, pre-established marital status categories in statistical reporting related to live births may distort the complexities and specificities of marriage arrangements of Indigenous mothers, who belong to diverse groups with social practices that may not be readily understood from a Western perspective. Third, some mothers may have had more than one live birth during the study period, however due to the lack of unique maternal identifiers in birth records, it is not possible to identify live births to the same mother. Lastly, there is the possibility of residual confounding in multivariate analyses due to the exclusion of potentially confounding variables such as paternal characteristics,^17^ or possible measurement errors in some variables. Despite these limitations, the results of this study are relevant for other countries in Latin America. 

## CONCLUSIONS

The prohibition of child marriage has contributed to the virtual disappearance of births to married mothers under age 18 in Ecuador between 2015 and 2020. Despite a significant reduction in births to minor mothers in stable unions, they continue to be prevalent. Our study confirms better reproductive health indicators among married adult mothers, but not necessarily among married minors. In contrast to what has been observed in Asia and Africa, single mothers in Ecuador account for the majority of births among minor mothers, with poorer health outcomes than those who are in stable unions. Therefore, prevention strategies for early pregnancy should contemplate the situation of single mothers under age 18 and seek to strengthen the autonomy of adolescents in terms of sexual and reproductive rights and family planning.
